# Supplementary material for: Improved cryo-EM reconstruction of sub-50 kDa complexes using 2D template matching
Source: eLife. 2026 Oct 26;15:RP109790. doi: 10.7554/eLife.109790 (PMC13707379; doi:10.7554/eLife.109790)
Supplement: Peer review history

# Editor report: eLife Assessment

**DOI:** 10.7554/eLife.109790.4.sa0

**Authors:** Sjors HW Scheres

**Affiliations:** MRC Laboratory of Molecular Biology United Kingdom

**Keywords:** Convincing, Important

This **important** study builds on previous work from the same authors to present a conceptually distinct workflow for cryo-EM reconstruction that uses 2D template matching to enable high-resolution structure determination of small (sub-50 kDa) protein targets. The paper provides **convincing** evidence that the density for small-molecule ligands bound to such targets can be reconstructed without these ligands being present in the template, and it provides an insightful quantification of the level of model bias that remains in the resulting reconstructions. The convenient visualisation of small molecules bound to protein targets of a known structure will be relevant for the pharmaceutical industry.

# Referee report: Reviewer #3 (Public review)

**DOI:** 10.7554/eLife.109790.4.sa1

Summary:

Due to the low SNR of cryo-EM micrographs necessitated by radiation damage, determining the structure of proteins smaller than 50 kDa is exceedingly challenging, such that only a handful have been solved to date. This work aims to improve the reconstruction of small proteins in single-particle cryo-EM by using high-resolution 2D template matching, an algorithm previously used to locate and align macromolecules in situ, to align and reconstruct small proteins. This approach uses an existing macromolecular structure, either experimentally determined or predicted by AlphaFold, to simulate a noise-free 3D reference and generates whitened projections, crucially including high-spatial-frequency information, to align particles by the orientation with maximal cross-correlation. They demonstrate the success of this approach by generating a 3D reconstruction from an existing dataset of a 41.3 kDa protein kinase that had previously evaded attempts at high-resolution structure determination. To alleviate concerns that this is purely from template bias, they demonstrate clear density at two regions that were not present in the template: 6 residues in an alpha helix and an ATP in the ligand binding pocket. The latter is particularly important for its implications in determining structures of ligand-bound proteins for drug discovery. They also produce a composite omit map from 36 partial-deletion reconstructions spanning the entire protein, demonstrating a reconstruction can be obtained without template bias. Additionally, the authors provide an update to the classic calculation in Henderson 1995 to predict the minimum molecular mass of a protein that can be solved by single-particle cryo-EM.

Strengths:

I am in no doubt that this technique can be used to gain valuable insights into the structures of small proteins, and this is an important advancement for the field. It is complementary to single-particle cryo-EM and provides an extra tool for the experimentalist that may work better in certain cases. For cases where only a small region of the structure is of interest, such as in drug screening, this method provides a simple workflow to screen many structures.

The claim that using high-spatial frequency information is essential for aligning small proteins is a valuable insight. A recent pre-print published at a similar time to this manuscript used high-resolution information in standard ab-initio reconstruction to generate a high-resolution reconstruction from the same dataset, supporting the claims made in the manuscript.

The theoretical section outlined in the appendix is also theoretically sound. It uses the same logic as Henderson, but applies more up-to-date knowledge, such as incorporating dose-weighting and altering the cross-correlation based noise estimation. This update is valuable for understanding factors preventing us from reaching the theoretical limit.

Weaknesses:

This method is a complementary technique to determine the structure of small macromolecules to existing methods such as Blush regularization and HR-HAIR. Although the authors have demonstrated convincingly that their method selects a stack of high-quality particles, it is less clear whether it performs better than RELION when using the same stack of particles, particularly in the ATP binding pocket. As the authors discuss, systematic benchmarks comparing these methods over more targets than the one presented here, will be important for determining the utility of this method.

The method presented here also introduces template bias. Omit maps are used to reduce template bias by removing the region of interest from the template. Producing a full reconstruction through a composite omit map is computationally expensive and can introduce artifacts at boundaries. Therefore, unless this method outperforms modern SPA methods, its major use case will likely be restricted to ligand binding studies rather than full 3D reconstructions.

# Author comment: Author response

**DOI:** 10.7554/eLife.109790.4.sa2

**Authors:** Kexin Zhang, Timothy Grant, Nikolaus Grigorieff

**Affiliations:** Howard Hughes Medical Institute Worcester, MA United States; University of Wisconsin-Madison Madison United States; Howard Hughes Medical Institute Worcester, MA United States

The following is the authors’ response to the previous reviews

**eLife Assessment**

The evidence described for the claim that this technique improves the alignment of the reconstruction of small complexes compared to standard techniques is incomplete. The authors could better evaluate the effects of model bias on the reconstructed densities, as suggested by reviewer #1.

We thank the editors for highlighting this remaining concern. To better evaluate the effects of model bias, we have performed the FSC-based analyses suggested by Reviewer 1, including a map–model FSC of the omit map and an FSC between the half-maps (though the latter is unreliable for the composite), and added the results to the revised manuscript (new Figure 5—figure supplements 1–3; detailed under Requested FSC Analysis below). We have additionally revised the text to avoid overstating the reconstruction as “unbiased” and to soften comparisons with other reconstruction methods (detailed below).

**Public Reviews:**

**Reviewer #1 (Public review):**

In the revised version, the refinement of atomic occupancies in the 2DTM-generated maps has been insightful: densities only come back at values ranging from 0.55–0.80, whereas residues included in the template remain at 1, suggesting that the 2DTM-reconstruction does suffer from model bias. Their newly added Omega calculations, which are helpful, also suggest that model bias is present in the 2DTM-based reconstructions. These observations therefore contradict the first subsection heading of the Results, which claims “unbiased reconstruction of omitted residues”.

We agree that the previous subsection heading was too absolute. We have changed the heading from “Unbiased reconstruction of omitted densities in a 43 kDa protein kinase” to “Recovery of densities omitted from the template in a 43 kDa protein kinase”. The opening sentence now states that we evaluated the ability of 2DTM to “recover omitted ligand densities” (L123–125).

We also revised nearby statements to describe the observations without claiming that the entire reconstruction is free of template bias. The manuscript now states that, because the corresponding features were omitted from the search template, the recovered densities cannot result from direct inclusion of those features in the template (L150–153).

For the omitted alpha-helical turn, we now state simply that its density was recovered despite its absence from the search template (L191–192). We also revised the interpretation of the occupancy-refinement results from “confirming partial, unbiased recovery” to “supporting partial recovery of density in the omitted regions” (L198–199). The same wording has been applied to the Supplementary file 1 caption on page 27.

Finally, the summary of the ligand-deletion experiments now states that a ligand and nearby residues can be deleted to reduce template bias while retaining sufficient signal for their density to be recovered (L248–251). In the Methods, we retain the description that the composite omit map was constructed to avoid template bias at the omitted locations (L953–954).

We have also toned down comparative statements about reconstruction accuracy, including the relevant subsection heading (“Comparison of 2DTM and RELION reconstructions from the same particle stack” at L252–253) and the surrounding discussion (L274–279).

Requested FSC Analysis

The measurement of how much model bias is present in this OMIT map by FSC calculations is still pending. This could be done in two ways. My original suggestion was to calculate a mapto-model FSC for the OMIT map and the full reference. This should be compared with a similar map-to-model FSC on the map where only the ligand was omitted. Alternatively, they can use the cisTEM FSC uncorr procedure on the OMIT half-reconstructions and compare the resulting curve with the one presented in Figure 1b.

We have now completed both analyses and added them to the revised manuscript (new Figure 5—figure supplements 1–3, with accompanying Results and Methods text).

(1) Map–model FSC. We computed the map–model FSC between the composite OMIT map and a density simulated from the full 1ATP model, and compared it with the equivalent FSC for the Figure 1 reconstruction, in which the ligand and residues 222–227 were omitted from the template (Figure 5—figure supplement 1). The composite OMIT map crossed FSC = 0.5 at 3.5 Å and FSC = 0.143 at 3.0 Å, compared with 3.0 Å and 2.4 Å, respectively, for the Figure 1 reconstruction. Because each local region of the composite map was taken from a reconstruction in which the corresponding residues were absent from the template, this agreement reflects genuine recovery rather than direct inclusion of those local features in the template. The lower FSC values relative to the Figure 1 reconstruction are expected. In the Figure 1 reconstruction, most of the protein remained in the template, whereas the composite is assembled from disjoint local omit regions. This stitched construction introduces holes and mask boundaries that affect Fourier-space agreement across the curve, in addition to the weaker, partial recovery of locally omitted density. Thus, the map–model FSC provides a conservative Fourier-space assessment of recovered omit-region density. The composite map–model FSC also shows a negative dip at the lowest spatial frequencies, which does not indicate failed recovery. Radial binning around the omitted atoms (Supplementary file 3) shows that the atom-centred shells (≤2 Å) recover positive but weakened density upon omission, whereas the peripheral shells (2–3 Å) are negative and nearly identical whether the residue is present or omitted, leading to net-negative density within the molecular envelope. We therefore interpret the low-frequency dip as a consequence of the composite construction rather than as evidence for failed recovery of omitted density.

(2) Half-map FSC_uncor_. We also computed the half-map FSC of the individual OMIT reconstructions (Figure 5—figure supplement 2): the 36 individual omit reconstructions cross FSC = 0.143 at a median resolution of 2.8 Å, comparable to the Figure 1b reconstruction (∼3.0 Å). The composite map’s own half-map FSC appears higher, but this value is not a reliable resolution estimate because the two composite half-maps are assembled using the same voxel-assignment masks. This shared support introduces artificial correlations, which we demonstrate with a phase-randomization control (Figure 5—figure supplement 3). We therefore use the individual omit-reconstruction FSCs and the composite map–model FSC, rather than the composite half-map FSC, to assess Fourier-space agreement.

Together, these analyses show that the OMIT reconstructions contain high-resolution signal in regions absent from the corresponding search templates, while also identifying the low-frequency dip and the inflated composite half-map FSC as consequences of the conservative stitched composite construction.

**Reviewer #3 (Public review):**

Nor was it compared to more recent strategies for processing SPA data from small molecules, such as Blush regularization or HR-HAIR. [...] This places this method as a complementary technique, and whether it outperforms those methods for a wide variety of molecules is yet to be determined.

We agree that a systematic comparison with recent small-particle SPA methods such as Blush regularization and HR-HAIR will be important. We have added this point to the Discussion (L831–846). We also note that such as comparison should consider not only particle stack and molecular mass, but also the fidelity of the 2DTM template forward model. In the ideal limit of an accurate template and forward model, 2DTM should provide a strong prior for particle detection and pose determination. In practice, however, current templates remain imperfect approximations to the experimental signal because of inaccurately modelled solvent-boundary effects and atomic scattering factors, bonding and charge redistribution, and conformational mismatch. Improving template generation is therefore an important direction for extending the range of molecular targets and imaging conditions where 2DTM can be applied.
